# Benefits of temporary alcohol restriction: a feasibility randomized trial

**DOI:** 10.1186/s40814-020-0554-y

**Published:** 2020-01-31

**Authors:** Matt Field, Jo-Anne Puddephatt, Laura Goodwin, Lynn Owens, Danielle Reaves, John Holmes

**Affiliations:** 1 Department of Psychology, University of Sheffield, Cathedral Court, 1 Vicar Lane, Sheffield, S1 2LT UK; 2 Department of Psychological Sciences, University of Liverpool, Liverpool, UK; 3 Institute of Translational Medicine, University of Liverpool, Liverpool, UK; 4 School of Health and Related Research, University of Sheffield, Sheffield, UK

**Keywords:** Alcohol, Cellular breathalyser, Temporary abstinence

## Abstract

**Background:**

Participation in temporary alcohol abstinence campaigns such as ‘Dry January’ may prompt enduring reductions in alcohol consumption. A randomized controlled trial (RCT) is required to establish any long-term benefits or negative consequences of temporary abstinence. In the present study, we randomized heavy drinkers to complete or intermittent alcohol abstinence for 4 weeks, in order to evaluate the feasibility of conducting a large-scale RCT.

**Methods:**

This was a mixed methods feasibility study in which we explored recruitment and retention to a randomized trial, compliance with alcohol abstinence instructions and barriers to compliance, and acceptability of study procedures (primary feasibility outcomes). A community sample of women aged between 40 and 60 who drank in excess of 28 alcohol units per week were randomized to abstain from alcohol for 4 weeks either completely or intermittently (at least four abstinent days per week). To monitor compliance, both groups provided regular breath samples on a cellular breathalyser. A subsample completed a semi-structured interview that probed barriers to compliance with abstinence instructions and acceptability of study procedures.

**Results:**

Within 5 months, we recruited, screened and randomized 25 participants (20% of participants who responded to advertisements: 14 in the complete abstinence group, 11 in the intermittent abstinence group), 24 of whom were retained throughout the 28-day intervention period. Participants in both groups tended to comply with the instructions: the median number of breathalyser-verified abstinent days was 24 (IQR = 15.5–25.0; 86% of target) in the complete abstinence group versus 12 (IQR = 10–15; 75% of target) in the intermittent abstinence group. Semi-structured interviews identified some barriers to compliance and methodological issues that should be considered in future research. No adverse events were reported.

**Conclusions:**

It is feasible to recruit heavy drinking women from community settings and randomize them to either complete or intermittent abstinence from alcohol for 4 weeks. The majority of participants were retained in the study and compliance with the abstinence instructions was good, albeit imperfect. A comprehensive RCT to compare temporary alcohol abstinence with other alcohol reduction strategies on long-term alcohol consumption is feasible. Findings from such a trial would inform implementation of alcohol campaigns and interventions.

## Background

Recent years have seen a surge in the popularity of organized campaigns in which alcohol consumers attempt to abstain from alcohol for a fixed period, typically 1 month. For example, an estimated four million people took part in ‘Dry January’ in the UK in 2018 [1], and similar campaigns are gaining traction worldwide, such as ‘Dry July’ in Australia. Among heavy drinkers, 1 month of abstinence from alcohol has beneficial effects on a number of indicators of physical health that are adversely affected by chronic heavy drinking including insulin resistance, blood pressure, body mass and cancer-related growth factors [2]. Furthermore, observational studies that followed up alcohol consumers 6 months after the temporary abstinence period demonstrated beneficial enduring effects, including less frequent drinking, a lower volume of alcohol consumption, increased confidence in the ability to resist alcohol (drinking refusal self-efficacy; DRSE) and a reduction in scores on the Alcohol Use Disorders Identification Test (AUDIT), such that participants were less likely to meet criteria for harmful drinking [2, 3].

Interpretation of findings from these observational studies is complicated by a number of factors. First, participants were a self-selected sample of alcohol consumers who either signed up to the Dry January campaign in the UK [3] or volunteered for a study that required participants to abstain from alcohol for 1 month [2]. Neither of these studies compared the effects of temporary abstinence with a different alcohol reduction strategy, such as attempting to cut down drinking rather than abstaining completely. Therefore, the observed reductions in alcohol consumption at follow-up could be attributed to participants’ motivation to reduce their drinking rather than the temporary abstinence period per se [4, 5], and comparable reductions in drinking might have been observed if participants had attempted to reduce their drinking in a different way [6]. In one study, alcohol consumers were randomized to either 3 weeks of complete abstinence versus 3 weeks of drinking alcohol as normal. This study demonstrated no group differences in self-reported alcohol consumption at follow-up 3 weeks later, which suggests that causal attributions of sustained reductions in drinking to temporary abstinence periods may be premature [7].

Second, as is standard with alcohol research, alcohol consumption during the temporary abstinence period and at follow-up was assessed with self-report in both of the observational studies [2, 3]. Self-reported alcohol consumption is influenced by impression management [8], and one might reasonably expect people who sign up to the Dry January campaign to feel pressured to claim that they are drinking less alcohol at follow-up. Furthermore, in the largest observational study [3], only 23% of the original participants could be recontacted at 6-month follow-up, and heavier drinkers were less likely to respond at follow-up. Therefore, the findings from this study [3] should be interpreted with caution because heavy drinkers (who might be relatively unlikely to reduce their drinking in the longer term) were underrepresented at follow-up.

In the UK, the Dry January campaign is marketed as ‘the perfect way to reset your relationship with alcohol’ [9]. The findings from observational studies [2, 3] are consistent with this claim, but the aforementioned methodological issues with these observational studies (self-selected samples, no control or comparison condition, follow-up data not missing at random) highlight the need for more rigorous research to evaluate the long-term benefits of temporary abstinence from alcohol. It is important to move beyond observational and cohort studies by conducting a randomized controlled trial (RCT) in which alcohol consumers are randomized to temporary abstinence versus control condition(s), and in which compliance with instructions is objectively verified rather than being reliant on self-reported alcohol consumption.

We plan to conduct such an RCT in which we will recruit heavy drinkers who are motivated to reduce their alcohol consumption and randomize them to either (a) 1 month of complete abstinence from alcohol, (b) 1 month of a different method of alcohol restriction, and (c) a further control condition in which participants continue to drink alcohol as normal. We will then assess alcohol consumption at follow-up. Comparison of the first and second treatment arms would permit an evaluation of whether complete abstinence from alcohol leads to larger or longer-lasting reductions in alcohol consumption compared with a less ‘all-or-nothing’ attempt at temporarily restricting drinking. Comparison of the first and second treatment arms with the third would permit an evaluation of the extent to which heavy drinkers who are motivated to change are likely to reduce their alcohol consumption in the absence of any attempt at temporary abstinence or drinking restriction. However, before conducting such an RCT, it is important to establish the feasibility of a number of aspects of the research methods.

Here we report a feasibility study in which we recruited heavy drinking women who were motivated to reduce their alcohol consumption and randomized them to either complete abstinence from alcohol for 4 weeks or intermittent abstinence (abstinence from alcohol for at least 4 days per week, every week) for the same period. Our target population was heavy drinking women aged between 40 and 60. We selected this population because alcohol consumption in this demographic is divergent from broader trends in the UK towards reduced drinking and abstinence [10–12]. Furthermore, participants who took part in an earlier evaluation of the Dry January campaign were primarily women, with a median age of 41 [3]. Our comparison condition was chosen on the basis of the UK Government recommendations that people who drink regularly and want to cut down should aim to completely abstain from alcohol on several days each week [13], advice that was recently reinforced by Drinkaware and Public Health England with the launch of their ‘Drink Free Days’ campaign in September 2018 [14].

We assessed participants’ compliance with the instructions (either to completely abstain from alcohol or to abstain on at least 4 days per week) by self-reports that were objectively verified by a cellular photo digital breathalyser, which is able to verify the identity of the person who provides the breath sample by taking their photograph at the same time as the breath sample (see [15, 16]). Soon after the end of the intervention period, a subset of participants completed a semi-structured interview which examined barriers to compliance with the abstinence instructions, and the acceptability of the research methods including the usability of study materials. Approximately 1 month after the end of the intervention period, participants were invited to attend a follow-up visit in which they reported their alcohol consumption and DRSE, alongside additional secondary outcome measures that may be included in a subsequent RCT.

The primary aims of this feasibility study were to establish:

- The feasibility of recruitment of heavy drinking women aged 40–60 who are motivated to reduce their alcohol consumption, from the local community;
- Participant retention throughout the intervention period and at 1-month follow-up;
- Compliance with abstinence instructions during the intervention period, and any barriers to compliance; and
- Acceptability of study procedures and usability of a smartphone alcohol monitoring app and cellular breathalyser.

## Methods

### Design and aims

This was a randomized feasibility study that employed quantitative and qualitative research methods to contrast two behaviour change interventions that prompt short-term reductions in alcohol consumption, in order to inform the feasibility of a subsequent randomized controlled trial that will evaluate their enduring effects on alcohol consumption, physical health and wellbeing. The two interventions were (i) complete abstinence from alcohol for 4 weeks and (ii) abstinence from alcohol for at least 4 days of the week, whilst being able to consume alcohol on the remaining days, also for 4 weeks. We ran the study over 5 months, between the beginning of February 2018 and the end of June 2018. The specific aims of this feasibility study were to understand the feasibility of recruitment and retention into the trial, quantify the extent of compliance with abstinence instructions and identify any barriers to compliance, and to probe the acceptability of the study procedures and usability of the study materials.

### Patient and public involvement (PPI)

Before designing the randomized feasibility study, we sought feedback from women in the local community who were representative of the target population. Volunteers were recruited to participate in informal focus groups via social media advertisements that requested women aged between 40 and 60 who were motivated to reduce their alcohol consumption. We ran two focus groups, both comprising five participants and a researcher who facilitated the discussion, in October and November 2017. Discussion topics included reasons for reducing alcohol consumption and the types of behavioural techniques used to cut down, perceptions of the short-term and long-term benefits and consequences of temporary abstinence campaigns such as ‘Dry January’, and the acceptability of different methods used for monitoring alcohol consumption, including self-reporting alcohol consumption via a smartphone app and direct monitoring of breath or blood alcohol concentrations using transdermal sensors (such as SCRAM CAM; www.scramsystems.com) and the Soberlink breathalyser (www.soberlink.com), which is described in detail below. The most important feedback from these focus groups was an interest in participating in a trial involving randomization to complete abstinence or a commitment to regular abstinent days for approximately 1 month, willingness to regularly report alcohol consumption using a smartphone app, and a preference for the Soberlink device rather than the SCRAM CAM or other transdermal alcohol sensors given the obtrusiveness of the latter.

### Setting

The study was conducted in the local community in, UK. Over the course of the intervention period, participants were required to attend testing sessions at the University of Liverpool campus, as detailed below.

### Participants—inclusion criteria

Women aged between 40 and 60 years of age, who reported drinking in excess of 28 units of alcohol per week (1 unit = 8 g alcohol; 28 units is double the UK ‘low risk drinking’ guideline amount of 14 units per week [13]), who were interested in reducing their alcohol consumption, and who had abstained from alcohol for at least two consecutive days within the past year. The final inclusion criterion was incorporated in order to exclude participants who may experience serious alcohol withdrawal symptoms if they were to completely abstain from alcohol.

### Exclusion criteria

Positive breath alcohol reading during screening; self-reported pregnancy; self-reported history of treatment for alcohol use disorder (including medical detoxification); self-reported alcohol consumption in excess of 10 units per day (based on average over previous month); moderate to severe alcohol dependence as inferred from a score of 15 or higher on the Severity of Alcohol Dependence Questionnaire [17]; possible alcohol-related physical comorbidity including history of self-reported diabetes, renal, liver, heart or lung disease. Some of these exclusion criteria (drinking in excess of 10 units per day, moderate or severe alcohol dependence) were applied in order to identify and exclude prospective participants who may experience serious alcohol withdrawal symptoms if they were to completely abstain from alcohol.

### Recruitment

Prospective participants were recruited via advertisements placed on social media targeted at people living in the local region (Merseyside, UK). Participants who responded to the advertisements were provided with information about the study, including the inclusion and exclusion criteria, before being invited to attend a screening visit on the University campus if they believed that they were eligible to take part.

### Interventions

Participants were instructed to either (a) completely abstain from alcohol for 4 weeks (‘complete abstinence’ group) or (b) abstain from alcohol for at least 4 days per week, whilst being able to consume alcohol on the remaining 3 days each week, also for 4 weeks (‘intermittent abstinence’ group).

### Randomization

Participants were randomized to conditions in a 1:1 ratio using a random number computer generator by block randomization; block sizes varied randomly between four and six. Participant allocations were sealed in numbered opaque envelopes that were opened by the researcher (JP) in the presence of the participant immediately before the beginning of the intervention period. The randomization was conducted by a different researcher (LG) who was independent of participant recruitment and testing.

### Outcome measures

In accordance with the CONSORT guidance for randomized pilot and feasibility studies [18], the primary outcomes were feasibility, acceptability and compliance outcomes. Secondary outcomes included those measures that may be included in any subsequent randomized controlled trial, as detailed below.

### Primary feasibility outcome measures

1. Feasibility of recruitment and retention of participants who meet the eligibility criteria. This was inferred from (a) the number of participants who agreed to take part as a percentage of participants who responded to advertisements and (b) the number of participants who returned to the University for a follow-up visit as a percentage of participants who remained in the study throughout the intervention period and were invited to attend follow-up.
2. Compliance with abstinence instructions during the study period, i.e. adherence to the intervention (the number of days of breathalyser-verified abstinence during the intervention period), and any barriers to compliance (based on semi-structured interviews).
3. Acceptability of the general study procedures, and usability of a smartphone app and cellular breathalyser (based on semi-structured interviews).

### Secondary participant-centred outcome measures

- The quantity and frequency of self-reported alcohol consumption at follow-up. This was included because, in an observational study, participants who temporarily abstained from alcohol reported reduced alcohol consumption at follow-up [3].
- Scores on the following questionnaires: Alcohol Use Disorders Identification Test (AUDIT [19]), Stages of Change Readiness and Treatment Eagerness Questionnaire (SOCRATES [20]) and Drinking Refusal Self-Efficacy Questionnaire (DRSEQ [21]). These questionnaires, which are well validated for use in the study population (adults who drink alcohol), were included because previous observational studies demonstrated changes in these questionnaires at follow-up after temporary abstinence from alcohol [2, 3].
- Performance on computerized Stop-Signal task [22] and Relevant-feature Stimulus–Response Compatibility (R-SRC) tasks [23]. These tasks are validated for use in the study population [22, 23], and they were included for exploratory purposes.
- Systolic and diastolic blood pressure, measured with an upper arm cuff. This was included because a previous study demonstrated reductions in both systolic and diastolic blood pressure after temporary abstinence from alcohol [2].

### Procedures

**Fig. 1:**
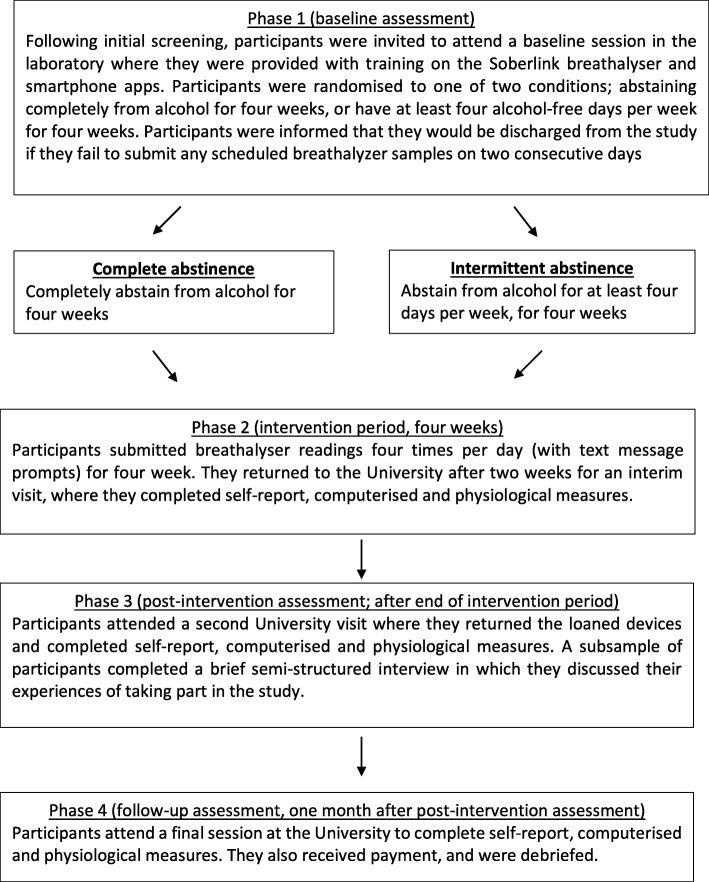
Schematic overview of study flow

A schematic overview of the study flow is shown in Fig. 1.

Participants who responded to advertisements were sent a detailed participant information sheet via email and invited to attend a screening appointment if they believed that they met the inclusion and exclusion criteria. Screening and all other University-based appointments took place in a private room on the University of Liverpool campus. Participants provided informed consent (for screening only; this included confirmation that their anonymized screening data would be included in reports), provided a breath alcohol sample and completed a questionnaire about their medical history including history of treatment for alcohol dependence/detoxification, before completing the Severity of Alcohol Dependence Questionnaire (SADQ) [17] and a 1-month timeline followback drinking diary (TLFB) [24].

Participants (*N* = 2) who met any of the exclusion criteria were advised to seek help from their GP or the Liverpool Community Alcohol Service, before being discharged and compensated (£10) for their time.

Participants who met all inclusion criteria then completed the Short-Form Health Survey (SF-12 [25]) and the Patient Health Questionnaire (PHQ-9 [26]) before completing the remainder of the secondary outcome measures (questionnaires, computerized tasks and blood pressure) as described above. They were then provided with a participant information sheet (for the full feasibility study), asked to carefully consider if they would like to take part and, if so, to book the next visit to the University at least 7 days later.

During this visit, participants provided informed consent before the researcher verified their identity, demonstrated how to use the Soberlink breathalyser (www.soberlink.com) and instructed participants how to use the Drinkaware app (https://www.drinkaware.co.uk/tools/app/) on a mobile phone that was loaned to them. The envelope containing the intervention allocation (complete abstinence or intermittent abstinence) was then unsealed, and participants’ instructions for the following 28 days were clarified by the researcher. Participants were instructed to provide scheduled breathalyser samples and transmit them four times per day and to record their alcohol consumption on the Drinkaware app at the end of each day. Participants were informed that if they failed to transmit any scheduled breathalyser samples for two consecutive days they would be discharged from the study.

During the 28-day intervention period, participants attempted to comply with their instructions regarding alcohol consumption (complete abstinence or intermittent abstinence) and to access the Drinkaware app at least once per day in order to record their alcohol intake. Automated text messages were sent to the participants four times per day, at the beginning of each scheduled breathalyser time window (8 am and 10 am, 12 pm and 2 pm, 5 pm and 7 pm, 10 pm and 12 pm). Each text message prompted participants to provide and transmit a breathalyser reading within the next 2 h.

Midway through the intervention period (after approximately 14 days), participants returned to the University for an interim visit where they completed a timeline followback drinking diary (using the information stored on their Drinkaware app) and the secondary outcome measures as described previously. Participants returned to the laboratory again as soon as possible after the end of the intervention period where they returned the breathalyser and mobile phone and completed a further timeline followback drinking diary and the secondary outcome measures. A subsample of participants (*N* = 20, the first 20 that completed the study) completed a brief semi-structured interview in which they discussed their experiences of taking part in the study including barriers to compliance with abstinence instructions, acceptability of study procedures and usability of the study materials. All participants were instructed to continue recording their alcohol consumption each day using the Drinkaware app for the following month (the follow-up period).

Approximately 1 month later, participants returned to the University for a follow-up visit in which they completed the secondary outcome measures again. Note that we did not attempt to follow-up the final seven participants who completed the intervention period and attended the post-intervention visit in mid to late June because those follow-up sessions would have fallen outside of the funding period (funding expired at the end of June 2018). At the end of the study, participants were debriefed and received financial compensation for their participation, which was contingent on the number of laboratory sessions attended (£20 per session, maximum four sessions (baseline, interim, post-intervention, follow-up)). Payments were not contingent on compliance with instructions or completion of scheduled breathalyser assessments.

### Sample size

We did not conduct a formal sample size calculation given that this was a feasibility study. In the broader literature, sample size recommendations for feasibility studies range between 12 and 35 per group [27, 28]. Therefore, we aimed to recruit 20 participants per group, although a constraint was that funding for the study was limited to 5 months (February to June 2018), so in practice, we aimed to recruit as many participants as possible within this period, subject to budgetary constraints and practicalities such as the number of breathalysers that could be loaned out to participants at any one time.

Semi-structured interviews were conducted with the first 20 participants who completed the intervention period.

### Data analysis

Given the small sample size, for the primary outcome measures, we report medians and interquartile ranges, supplemented with nonparametric tests to explore group differences and changes over time (within groups). Results from these hypothesis-driven tests should be interpreted with caution given that our study was underpowered. Transcripts from the semi-structured interviews were analysed using inductive thematic analysis which permits themes and codes to be strongly linked to the data [29]. This method involves a five-phase approach: (a) familiarization with the data, (b) generating initial codes, (c) searching for themes, (d) reviewing themes and (e) defining and naming themes [29]. NVivo 10 [30] was used to facilitate the coding process, and the analysis continued in an iterative process whereby raw data was continually analysed to identify themes (which could be merged, removed or stratified if redundant). A sample of extracted data representative of key codes and themes was sent to a second coder (DR) along with a developed codebook to establish reliability. The primary coder (JP) reviewed coded extracts to establish the consistency of coding, and any disagreements were resolved through discussion.

## Results

### Recruitment and retention

**Fig. 2:**
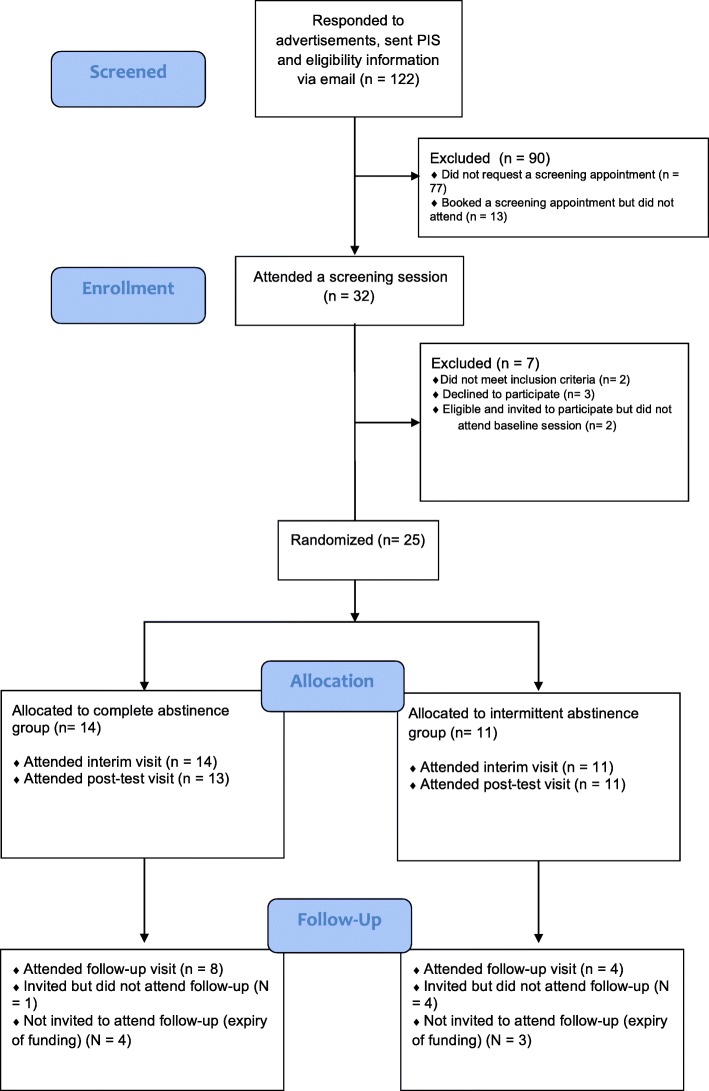
CONSORT extension for randomized pilot and feasibility trial flow diagram

See Fig. 2 for CONSORT flowchart. Of 122 participants who responded to advertisements, 32 booked and attended a screening appointment, of whom 25 were eligible for and agreed to participate in the study and were randomized to a condition. Therefore, 20% of the participants who responded to advertisements were randomized. Of the 25 participants who were randomized, the majority (24; 96%) completed the intervention period. One participant in the complete abstinence condition withdrew from the study immediately after the interim assessment. Regarding participant retention at follow-up, note that we did not invite the final seven participants to attend the follow-up appointment (as this would have fallen outside the funding period). Of 17 participants who completed the intervention period and were invited to attend a follow-up session, 12 attended, resulting in a follow-up rate of 71%.

### Participant characteristics

Participant characteristics at the screening assessment, for both experimental groups and for those participants (*N* = 7) who were not randomized, are shown in Table 1. The majority of participants were White British and university educated. Scores on the AUDIT indicated hazardous or harmful drinking or probable alcohol dependence in all participants, although physical alcohol dependence (inferred from the SADQ) was mild, on average. Participants’ alcohol consumption was well in excess of the UK low-risk drinking guidelines of 14 units (112 g of alcohol) per week. However, most participants did not drink alcohol every day.

**Table 1:** Participant characteristics (recorded at screening assessment). Values are median (interquartile range) unless stated

Variable	Complete abstinence group (*N* = 14)	Intermittent abstinence group (*N* = 11)	Not randomized (*N* = 7)
Age (years)	50.50 (43.75–54.00)	46.00 (44.00–50.00)	50.00 (45.00–56.00)
Ethnicity (% White British)	85.71%	90.91%	100%
SES (% with university degree)	71.43%	81.82%	71.43%
Body mass index	28.40 (24.53–33.88)	25.50 (24.10–34.70)	28.60 (22.10–35.90)
SF-12 total	39.50 (38.00–41.50)	38.00 (31.00–40.00)	36.00 (30.00–41.00)
PHQ-9 total	2.00 (1.00–3.25)	3.00 (2.00–6.00)	7.00 (4.00–10.00)
Alcohol consumption (grams per week)	263.00 (209.50–342.50)	302.00 (225.00–345.00)	320.00 (228.00–504.00)
Drinking days per week	3.88 (3.44–5.06)	4.00 (3.25–6.50)	6.75 (5.50–6.75)
AUDIT total	14.50 (11.50–23.75)	12.00 (11.00–19.00)	17.00 (14.00–28.00)
SADQ total	6.50 (3.75–10.50)	3.00 (3.00–11.00)	4.00 (3.00–7.00)
DRSEQ social pressure	2.83 (1.67–3.00)	3.00 (2.33–4.33)	3.33 (1.50–4.00)
DRSEQ emotional relief	3.33 (3.00–4.08)	3.67 (2.33–4.67)	2.00 (1.33–3.33)
DRSEQ opportunistic	4.67 (3.58–5.00)	5.00 (4.00–5.67)	3.00 (2.33–4.33)
SOCRATES Recognition	18.50 (15.75–22.25)	18.00 (12.00–23.00)	22.00 (18.00–31.00)
SOCRATES Ambivalence	11.50 (10.00–14.00)	12.00 (10.00–14.00)	13.00 (12.00–19.00)
SOCRATES Taking steps	27.00 (20.75–33.25)	21.00 (17.00–26.00)	22.00 (17.00–30.00)

*SF12* Short-Form Health Survey, *PHQ-9* Patient Health Questionnaire, *AUDIT* Alcohol Use Disorders Identification Test, *SADQ* Severity of Alcohol Dependence Questionnaire, *DRSEQ* Drinking Refusal Self-Efficacy Questionnaire, *SOCRATES* Stages of Change Readiness and Treatment Eagerness Questionnaire

### Compliance with abstinence instructions

Data are shown in Table 2. Participants in the complete abstinence group should have abstained from alcohol on all 28 days of the intervention period, whereas the target for participants in the intermittent abstinence group was 16 days of abstinence (4 days per week, for 4 weeks). Overall, participants in both groups managed to comply with instructions on the majority of days: the median number of breathalyser-verified abstinent days was 24 in the complete abstinence group (86% of the target 28 days), versus 12 in the intermittent abstinence group (75% of the target 16 days).

**Table 2:** Compliance with instructions. Values are medians (interquartile range)

	Complete abstinence group	Intermittent abstinence group
	(*N* = 13)	(*N* = 11)
Self-reported abstinent days	25 (21–27.5)	16 (15–18)
Breathalyser-verified abstinent days	24 (15.5–25)	12 (10–15)
% of self-reported abstinent days that were breathalyser-verified	92.31 (75.60–96.08)	78.95 (62.5–88.89)
Self-reported alcohol consumption on drinking days (grams)	56.35 (37.86–67.73)	61.60 (53.51–78.67)
BAC on positive breathalyser tests (% BAC)	.06 (.03–.08)	.06 (.04–.09)

There were statistically significant group differences in self-reported abstinent days (Mann-Whitney *U* = 1, *Z* = − 4.10, *p* < .001), breathalyser-verified abstinent days (Mann-Whitney *U* = 18.50, *Z* = − 3.09, *p* < .002), and the percentage of self-reported abstinent days that were breathalyser-verified (Mann-Whitney *U* = 37.50, *Z* = − 1.97, *p* = .047), all of which were higher in the complete abstinence compared with the intermittent abstinence group. However, group differences in self-reported alcohol consumption on drinking days (Mann-Whitney *U* = 34.50, *Z* = − 1.45, *p* = .15) and breath alcohol content on drinking days (Mann Whitney *U* = 36.00, *Z* = − 1.03, *p* = .33) were not statistically significant.

### Compliance with scheduled breathalyser assessments

Data are shown in Table 3. There were no significant group differences in the number of days with at least one missing scheduled breathalyser assessment (Mann-Whitney *U* = 61.50, *Z* = −.58, *p* = .56). Regarding the fine-grained breathalyser data, the intermittent abstinence group returned more positive breath alcohol samples than the complete abstinence group at the third (Mann-Whitney *U* = 22.5, *Z* = − 3.00, *p* = .003) and fourth (Mann-Whitney *U* = 14.50, *Z* = − 3.33, *p* = .01) scheduled assessments of each day (note that these contrasts were not corrected for multiple comparisons). All other contrasts were not statistically significant (*p* > .1).

**Table 3:** Characterization of breathalyser tests. Values are medians (interquartile range). *BAC* breath alcohol content

	Complete abstinence group (*N* = 13)	Intermittent abstinence group (*N* = 11)
Number of days with at least one missed breathalyser assessment	6 (2–17)	9 (5–12)
First assessment (8 am–10 am)		
% of scheduled breathalyser tests with positive BAC	0 (0–0)	0 (0–19)
% of scheduled breathalyser tests with zero BAC	96 (85–100)	93 (78–96)
% of scheduled breathalyser tests not completed (BAC missing)	4 (0–13)	4 (4–11)
Second assessment (12 pm–2 pm)		
% of scheduled breathalyser tests with positive BAC	0 (0–4)	0 (0–4)
% of scheduled breathalyser tests with zero BAC	93 (70–96)	78 (74–85)
% of scheduled breathalyser tests not completed (BAC missing)	7 (0–30)	15 (11–26)
Third assessment (5 pm–7 pm)		
% of scheduled breathalyser tests with positive BAC	0 (0–4)	11 (7–14)
% of scheduled breathalyser tests with zero BAC	86 (75–96)	82 (68–89)
% of scheduled breathalyser tests not completed (BAC missing)	14 (4–18)	11 (4–18)
Fourth assessment (10 pm–12 am)		
% of scheduled breathalyser tests with positive BAC	7 (0–14)	25 (21–32)
% of scheduled breathalyser tests with zero BAC	75 (55–95)	57 (54–75)
% of scheduled breathalyser tests not completed (BAC missing)	14 (4–27)	11 (7–14)

### Semi-structured interviews

**Fig. 3:**
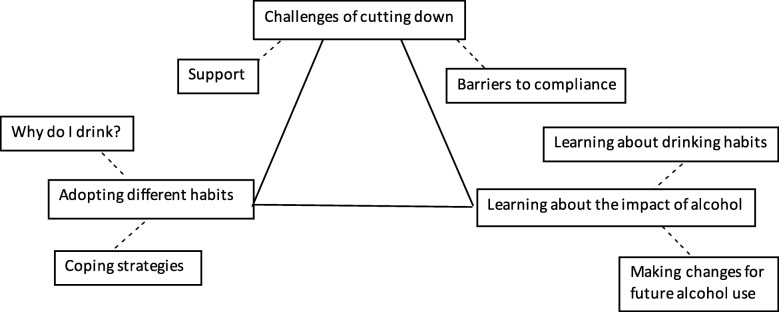
Developed thematic map illustrating qualitative analysis of transcripts from semi-structured interviews

Of those interviewed, 11 participants were in the complete abstinence group and nine were in the intermittent abstinence group. Our thematic analysis identified three main themes: (1) ‘challenges of cutting down’, (2) ‘adopting different habits’ and (3) ‘learning about the impact of alcohol’ (Fig. 3).

#### Theme 1: Challenges of cutting down

The majority of participants had not previously attempted to abstain from alcohol, and many discussed how challenging this was, particularly those allocated to the complete abstinence condition. This theme comprised two sub-themes: ‘barriers to compliance’ and ‘support’.

##### ‘Barriers to compliance’ (sub-theme)

Difficulty in abstaining from alcohol was often attributed to external factors such as being in the presence of others who were drinking alcohol:

… it was like a really hot sunny day, sitting in the beer garden and everyone else’s drinking … cider with loads of ice and I was like, “Oh yeah, have one of them.” (Participant 18)

Some participants tried to switch to soft drinks or alcohol-free alternatives, but they did not find these sufficiently enjoyable:

I bought some alcohol free sparkling wine … It’s nice. It’s obviously not...not the same as wine (Participant 23)

##### ‘Support’ (sub-theme)

Social support was highlighted as playing an important role in encouraging compliance with abstinence instructions. Conversely, the absence of support was implicated in difficulty abstaining:

… a supportive partner. She didn’t stop drinking, but there was no fuss made about it. We’re very sociable with our neighbours they didn’t stop drinking, but they didn’t make any fuss about it whatsoever … (Participant 13)

… everybody else was like, “go on, go on, go on, everybody else will be having a drink today. … And in the end, I cracked and I got a bottle of fizzy wine. (Participant 23)

Some participants reported that the requirement to submit regular breathalyser readings helped them to comply with abstinence instructions, and therefore, this functioned as a form of external support:

I found the fact that I was having to breathalyse myself four times a day um, made it easier to abstain. Knowing I was almost answerable to the breathalyser. (Participant 1)

… people would go “Oh, just one wouldn’t hurt,” … whereas this, this time you’re like, “No, I’m on a study and I’ve got breathalyser and I can’t cheat, it’s all being recorded,” so. And I think it got rid of the peer pressure aspect, um, which just makes life a bit easier really. (Participant 9)

#### Theme 2: Adopting different habits

Participants reported that a significant change in lifestyle was required if they were to comply with their abstinence instructions. They reflected on their previous drinking habits and motives for drinking in order to adapt to a new routine and learn new strategies. Alternative behaviours needed to be as rewarding as alcohol, hence the inclusion of two sub-themes: ‘why do I drink?’ and ‘coping strategies’.

##### Why do I drink?

Participants reflected on their entrenched drinking habits and their reasons for drinking. Some participants reported drinking out of habit at the end of the working day, in social circumstances or to cope with negative mood. Others drank because they enjoyed the taste and ‘feel’ of alcohol, for example:

I think that’s a lot of the problem, is that when I’m drinking on an evening, I’m drinking not just because it’s alcohol but because I actually like the taste of it. (Participant 9)

Once participants understood this, they often considered alternative behaviours that had the potential to be as rewarding as alcohol. This was particularly difficult for the complete abstinence group and hence relates to the ‘challenges of cutting down’ theme. The following quote is from a participant who tried alcohol-free drinks at the beginning of the study, but subsequently struggled to continue with this routine as those drinks did not help them to relax in the same way that alcohol did:

Friday night’s the worst one for me ‘cause get home, I’ve had a full week in [work] and get to Friday night, it’s like I just wanna sit down, have a glass of wine and go to bed … makes it sound like some sort of desperate alcoholic who’s desperate … to have a drink. But you know it’s...it’s just nice, isn’t it, to relax? (Participant 23)

##### Coping strategies (sub-theme)

Participants reported using a variety of coping strategies, particularly behaviour substitution which involved replacing alcohol with unhealthy food, alcohol-free or soft drinks. Some participants, particularly those in the complete abstinence condition, found alcohol-free drinks a useful alternative:

I still went out and I still socialised. Um, but I actually bought in alcohol-free substitutes, so if the temptation was there, then I would … have a drink and I’d feel like I was actually doing something. (Participant 1)

Several participants reported ‘treating themselves’ to unhealthy food in the absence of alcohol, particularly if they had complied with abstinence instructions:

I ate more salted snacks … I probably ate more because I think that was my treat to myself because I was, you know, doing the abstinence (Participant 13)

Participants in the intermittent abstinence condition reported planning their allowed ‘drinking days’ in advance, for example:

So I looked at … knowing what my social life was doing. Um, and obviously I’ve saved my three days for any social activities that I had. So they were more or less you know I didn’t really have much leeway. (Participant 15)

#### Theme 3: Learning about the impact of alcohol

Several participants discussed how the study enlightened them about the amount of alcohol they normally consume, the alcohol and calorie content of different drinks, and provided insights into how they might reduce their drinking after completing the study. There are two sub-themes: ‘learning about drinking habits’ and ‘making changes for future alcohol use’.

##### Learning about drinking habits (sub-theme)

In planning their abstinent days, some participants in the intermittent abstinence condition realized that they had developed a habit of near daily drinking:

It broke a habit because I’d found I would have a bottle of wine start it one night, not finish it. So, I’ll have a glass the next night and carry it forwards. So, I wasn’t drinking enormous quantities every night but I found that I was drinking most nights because I was always finishing off a bottle or something like that. (Participant 2)

The Drinkaware app was perceived as providing useful feedback about alcohol intake and calories from alcohol:

It was a bit scary when I actually converted it into calories. … when I put the five in (Drinkaware app), on the Friday night, it was like 1,200 calories. (Participant 3)

Some participants, particularly those in the complete abstinence condition, reported health benefits that they attributed to reduced alcohol consumption. Improvements in sleep quality, weight loss and productivity were most frequently mentioned, for example:

… I actually felt physically much better and I was sleeping a lot better so I had more energy. Much more energy. (Participant 13)

I’d happily do that experiment for the rest of my life. And it would probably made me live far longer and far happier and far healthier (Participant 9)

Some participants reported discussing the impact of alcohol and calorific information of alcohol with peers and colleagues suggesting the wider implications on participants’ social networks:

One of my friends said she took out about eight miniatures with her. And they were all gone as well as all the drinks. So I said “Well, that’s, you know, 16 drinks … 16 units besides.” So it’s a lot, isn’t it? (Participant 16)

Many participants reported that committing to the study, attempting to make changes in their alcohol consumption and using coping strategies helped them to realize that they were capable of cutting down drinking without making substantial sacrifices:

I would always, perhaps more often than not, have a glass of wine and I know that I can do without that now … filling in the apps kind of made me not want to drink to silly excess like binge drinking really at the weekend … it’s definitely made me re-evaluate the amount that I drink (Participant 12)

I think I used alcohol as a bit of a reward. Whereas I realise now that I don’t need to say at the end of a … not a stressful day … but at the end of the day I’ll relax and I’ll have a drink. Now I realise that I can have a treat in another way. (Participant 13)

##### Making changes for future alcohol use

Many participants reported intending to sustain changes in their alcohol consumption as a result of learning about their drinking habits. In particular, participants intended to continue having alcohol-free days after completing the study, for example:

I might only drink one day, and that’s again related to usually social occasions, so the other ones where I’m just sort of maybe randomly having one in the house, they don’t really bother me, and the other ones where they’re probably just a waste of calories and alcohol intake. (Participant 19)

I’m gonna keep it up Monday through Thursday, abstaining and then just having a drink at the weekend (Participant 23)

Other participants reported intending to continue using the Drinkaware app and other tools to maintain this change in behaviour:

I’ve also ordered a measure off EBay, so that if I … when I go back to drinking, because I tend to drink gin, um, I’ll measure my … my measures to make sure that they are … I do try and stay within my … my daily units. (Participant 1)

#### Acceptability of study procedures and usability of study materials

The majority of participants were in employment and several had children; therefore, it was important that the study procedures could fit into their normal routine. In particular, the requirement to submit four breathalyser readings per day, each within a specified and inflexible time window, was mentioned as unduly burdensome by some participants:

Maybe three. And maybe like breakfast, dinner, if you could do it with your food (Participant 5)

Possibly it could be more flexible around the different people’s lifestyles but I suppose it depends what you’re trying to achieve. (Participant 7)

Participants were loaned a mobile phone with the DrinkAware app preinstalled; this phone also received the reminder text messages. Some participants found it burdensome to carry this mobile phone alongside their own mobile phone, to the extent that they did not carry it around with them and therefore they could not receive reminder text messages (they installed the DrinkAware app on their own phone instead). This may partially explain why compliance with scheduled breathalyser readings was poor in some participants:

I’ve got a work phone. I’ve got a personal phone. I set an alarm on my personal phone to just remind me. So I didn’t even look at the [loaned] phone to be honest after the first day of getting it. (Participant 15)

Although many participants reported that feedback on drinking provided by the DrinkAware app was useful (see above), those in the complete abstinence condition accessed the app less frequently, and therefore they found it less useful:

I felt that the other group, I appreciate that there’s two groups, so there’s another group um, well I drink up to three times a week, so they would be actually monitoring how much alcohol, how many … how many units they were having. So I’m sure the … the Drinkaware app would’ve been a very valuable tool to them, whereas mine for myself it’s just it wasn’t a great deal of anything (Participant 1)

### Secondary outcomes

These findings are reported in Additional file 1. Self-reported alcohol consumption at follow-up was lower compared with baseline in the complete abstinence group, but there was no change in the intermittent abstinence group. The converse pattern was seen for (emotional) drinking refusal self-efficacy, which was improved at follow-up compared with pre-intervention in the intermittent abstinence group only. Both of these findings should be interpreted with caution given the small number of participants who were retained at follow-up (*N* = 8 in the complete abstinence group versus *N* = 4 in the intermittent abstinence group), and because all between-group differences were not statistically significant.

### Adverse events

No adverse events were reported.

## Discussion

Within 5 months, we were able to recruit and randomize 25 participants (~ 20% of those who responded to advertisements), the majority of whom (96%) were retained throughout the intervention period. The majority of participants in both groups tended to comply with their abstinence instructions. This resulted in a reliable difference between the complete and intermittent abstinence groups: the median number of breathalyser-verified abstinent days was 24 in the complete abstinence group (86% of the target 28 days) versus 12 in the intermittent abstinence group (75% of the target 16 days). Furthermore, our qualitative analysis revealed a number of barriers to compliance with abstinence instructions and some minor issues with study procedures that might be modified in order to improve the feasibility of a larger trial.

Two of the primary aims of this study were to understand the feasibility of recruiting heavy drinking women aged between 40 and 60 into a trial of this type and to estimate retention of participants in the trial throughout the intervention period and subsequent follow-up. We were able to recruit 25 participants within a 5-month period and to retain the majority of those (96%) in the study throughout the intervention period. In addition, 71% of participants, who were invited to attend a follow-up assessment 1 month after the end of the intervention period, did so. Overall, these figures on recruitment and retention of this population compare favourably to those from existing studies on interventions to reduce harmful and hazardous drinking in participants recruited from community settings (of which there are few, but see [31–33]), and they are encouraging regarding the feasibility of conducting a larger trial.

Regarding participants’ compliance with the instructions (to either completely abstain from alcohol or to abstain from alcohol for at least 4 days per week), we noted that participants in the complete abstinence group completely abstained from alcohol on the majority of days during the 28-day intervention period (median = 24 days), despite receiving no financial or other incentive to do so. Similarly, participants in the intermittent abstinence group abstained on 12 days on average, over the course of the intervention period, compared with a target of 16 days (4 days per week). These compliance findings are encouraging regarding the feasibility of a larger trial, although they highlight the need to consider ways to maximize compliance with the instructions, particularly in the complete abstinence group.

The thematic analysis of semi-structured interviews that were conducted with a subset of participants (*N* = 20) who completed the intervention sheds some light on barriers to compliance with abstinence instructions and the acceptability of the study procedures, and how these might be overcome in a future trial. Participants reported difficulty in abstaining from alcohol when they were in situations in which they typically drank and when they experienced social pressure to drink alcohol, and when alcohol-free drinks were perceived as unappealing. By contrast, participants attributed success at complying with abstinence instructions to social support, continuous self-monitoring, careful planning of social activities that were likely to involve drinking, and consumption of alcohol-free drinks. This accords well with previous literature on changes to social rituals that are associated with reduction of alcohol consumption [34] and with behaviour change techniques such as behaviour substitution, goal setting and self-monitoring that prompt change in drinking after other interventions [35, 36]. Furthermore, many participants perceived health benefits of abstaining from alcohol, as has been reported elsewhere [37], and they reported gaining a better understanding of their drinking and how habitual daily drinking had become. These benefits of abstinence and the techniques that participants reported using to help them to comply with abstinence instructions could be highlighted to participants in any future trial, in order to maximize compliance with abstinence instructions.

Regarding participants’ experience of the study procedures, there were no reported problems with the cellular breathalyser, although some participants objected to the requirement to carry an additional mobile phone with them, and to the frequency of scheduled breathalyser assessments. In addition, participants in the complete abstinence condition objected to the requirement to access the Drinkaware app every day in order to record their alcohol consumption, given that they were reporting ‘zero’ on the majority of study days. It would be desirable to use this feedback to increase the acceptability of study procedures in any future trial. For example, all apps could be installed on participants’ own phones, the first scheduled breathalyser assessment of the day might be dropped (given that participants submitted a negative reading on the first scheduled breathalyser assessment on the majority of days), and reporting of abstinent days could be made easier for participants. However, if changes are made to study procedures, it will be important to ensure that procedures remain matched across conditions, including in any additional comparison or control conditions, in order to standardize potentially confounding variables including the extent of self-monitoring of alcohol consumption.

A notable limitation of our study is that we had a limited budget and timeframe in which to complete the project, which meant that we were unable to recruit the number of participants that we intended to, and we were also unable to invite all participants who completed the intervention to attend a follow-up session. In addition, caution is required before generalizing our findings from a sample of heavy drinking women aged between 40 and 60 to other demographic groups; further work is required to characterize the feasibility of the study procedures in the broader population of alcohol consumers. Our ‘complete abstinence’ condition should not be seen as directly analogous to participation in organized temporary abstinence campaigns such as Dry January because it lacks the social characteristics, particularly ‘social contagion’, of those campaigns [37]. In addition, the requirements for participants to regularly engage with the DrinkAware app [38] and to submit regular biochemical verification of their abstinence [39] may have functioned as a powerful alcohol intervention in itself; therefore, one should not assume that the compliance rates reported here would generalize to all participants who attempt to temporarily abstain from alcohol. Any larger-scale effectiveness study will need to carefully balance the importance of obtaining accurate measurements of participants’ alcohol consumption (which may require biochemical verification) with the recognition that implementation of these interventions at the population level cannot realistically be combined with biochemical verification. Finally, we reported hypothesis-driven tests to explore group differences in primary feasibility outcomes (compliance with abstinence instructions and with scheduled breathalyser assessments), but results from these tests should be interpreted with caution given that that they are not recommended for use in small underpowered feasibility studies [18].

Our study also has strengths, particularly the use of cellular breathalysers to verify participants’ self-reported alcohol abstinence, which overcomes one of the major weaknesses of observational studies of the effects of self-reported temporary abstinence from alcohol (e.g. [3]). Most importantly, many of our participants reported numerous benefits of abstaining from alcohol and paying attention to and trying to change their drinking behaviour, as has been reported elsewhere [3, 37]. This suggests that campaigns such as Dry January and ‘Drink Free Days’ have an important role to play in helping heavy drinkers to reduce their alcohol consumption, which highlights the importance of rigorously evaluating the effects of temporary abstinence on alcohol consumption in the longer term.

## Conclusions

This feasibility study succeeded in recruiting 25 heavy drinking women from the local community who agreed to be randomized to either complete or intermittent abstinence from alcohol for 4 weeks. The majority of participants completed the intervention period, and compliance with abstinence instructions was good, albeit imperfect. Overall, these findings suggest that a large-scale RCT is feasible, and our qualitative findings suggests ways in which the study procedures might be adapted in order to maximize compliance with instructions, participant retention and acceptability of study procedures.

## Additional file

**Additional file 1: Table S1** Self-reported alcohol consumption at baseline and follow-up in participants who attended follow-up. Values are medians (IQR in brackets). **Table S2** Changes in DRSE over time. Values are medians (IQR) (N). **Table S3** Additional secondary outcome measures over time. Values are medians (IQR) (N)

## Data Availability

Anonymized raw data and a syntax file are available in SPSS format at https://osf.io/6q87x/
