# Supplementary material for: Benefits of temporary alcohol restriction: a feasibility randomized trial
Source: Pilot Feasibility Stud. 2020 Jan 31;6:9. doi: 10.1186/s40814-020-0554-y (PMC6995140; doi:10.1186/s40814-020-0554-y)
Supplement: Supplementary file 1 — Additional file 1: Table S1 Self-reported alcohol consumption at baseline and follow-up in participants who attended follow-up. Values are medians (IQR in brackets). Table S2 Changes in DRSE over time. Values are medians (IQR) (N). Table S3 Additional secondary outcome measures over time. Values are medians (IQR) (N) [file 40814_2020_554_MOESM1_ESM.docx]

**Supplementary results**

**Alcohol consumption at follow-up (Table S1)**

There were no significant between-group differences in alcohol consumption at baseline or follow-up (p > .1). However, within-subject contrasts, performed separately on each group, revealed a significant reduction in self-reported weekly alcohol consumption (in grams) from baseline to follow-up in the complete abstinence group only (Wilcoxon’s test, (z = -1.96, p = .05). This contrast was not significant in the intermittent abstinence group, and none of the other within-subject contrasts on any of the other variables were statistically significant (p > .1).

**Drinking-refusal self-efficacy (DRSE) (Table S2)**

There were no significant between-group differences at baseline, post-test or follow-up (p > .1). Within-subject contrasts, performed separately on each group, revealed significant improvements in emotional DRSE between baseline and post-test (Wilcoxon’st test, z = -2.46, p = .014) and a trend for further improvements between post-test and follow-up (z = -1.83, p = .07) in the intermittent abstinence group only. All other contrasts were not statistically significant (p > .1).

**Other outcome measures (Table S3)**

Descriptive statistics for the AUDIT and SOCRATES questionnaires and blood pressure are shown in Table S3. There were some technical problems with administration of the computerized Approach-Avoidance and Stop-Signal tasks which meant that very few participants completed these tasks at both baseline and post-test assessments. Specifically, only three participants completed the Approach-Avoidance task at both baseline and post-test: two in the complete abstinence group and one in the intermittent abstinence group. Only eight participants completed the Stop-Signal task at both baseline and post-test (four from each group). Given the high rates of missing data from these tasks, descriptive statistics are not reported.

Table S1: Self-reported alcohol consumption at baseline and follow-up in participants who attended follow-up. Values are medians (IQR in brackets)

Complete abstinence group (N = 8) Intermittent abstinence group (N = 4)

Alcohol consumption (g per week)

Baseline 241.00 (212.75 – 280.00) 317.00 (238.75 – 366.00)

Follow-up 119.00 (78.50 – 119.00) 213.00 (108.50 – 295.00)

Drinking days per week

Baseline 3.75 (3.50 – 4.44) 4.63 (4.00 – 6.19)

Follow-up 3.00 (1.13 – 3.75) 3.88 (2.69 – 5.06)

Alcohol consumed on drinking days (grams)

Baseline 61.56 (54.93 – 76.71) 62.18 (56.42 – 69.36)

Follow-up 45.03 (29.64 – 72.40) 52.00 (32.43 – 77.46)

Table S2: Changes in DRSE over time. Values are medians (IQR) (N)

Complete abstinence group Intermittent abstinence group

Social pressure

Baseline 2.67 (1.67 – 3.00) (N = 13) 3.00 (2.33 – 4.33) (N = 11)

Post-test 3.33 (2.17 – 4.00) (N = 13) 3.67 (3.00 – 4.67) (N = 11)

Follow-up 4.17 (3.42 – 4.83) (N = 8) 3.67 (1.75 – 6.33) (N = 4)

Emotional relief

Baseline 3.33 (3.00 – 4.17) (N = 13) 3.67 (2.33 – 4.67) (N = 11)

Post-test 4.33 (2.67 – 4.33) (N = 13) 4.67 (3.67 – 6.67) (N = 11)

Follow-up 4.67 (3.17 – 5.33) (N = 8) 2.83 (1.33 – 5.33) (N = 4)

Opportunistic

Baseline 4.67 (3.50 – 5.00) (N = 13) 5.00 (4.00 – 5.67) (N = 11)

Post-test 4.33 (3.50 – 6.17) (N = 13) 5.67 (5.00 – 5.67) (N = 11)

Follow-up 5.67 (4.25 – 7.00) (N = 8) 5.50 (5.08 – 5.92) (N = 4)

____________________________________________________________________________________________________________________

Response options range from 1 to 7, higher values indicate greater drinking refusal self-efficacy (DRSE)

Table S3: Additional secondary outcome measures over time. Values are medians (IQR) (N)

Complete abstinence group Intermittent abstinence group

AUDIT

Baseline 14.00 (11.00 – 24.50) (N = 13) 12.00 (11.00 – 19.00) (N = 11)

Post-test 11.00 (7.50 – 15.00) (N = 13) 11.00 (9.00 – 13.00) (N = 11)

Follow-up 11.50 (7.75 – 13.75) (N = 8) 17.00 (11.50 – 21.75) (N = 4)

SOCRATES Recognition

Baseline 19.00 (15.50 – 22.50) (N = 13) 18.00 (12.00 -23.00) (N = 11)

Post-test 13.00 (10.50 – 25.00) (N = 13) 16.00 (11.00 – 18.00) (N = 11)

Follow-up 12.50 (10.25 – 23.75) (N = 8) 20.50 (16.50 – 32.75) (N = 4)

SOCRATES Ambivalence

Baseline 11.00 (10.00 – 14.00) (N = 13) 12.00 (10.00 – 14.00) (N = 11)

Post-test 9.00 (6.00 – 14.50) (N = 13) 10.00 (7.00 – 13.00) (N = 11)

Follow-up 7.00 (4.25 – 13.50) (N = 8) 14.00 (11.50 – 18.00) (N = 4)

SOCRATES Taking steps

Baseline 24.00 (20.50 – 33.50) (N = 13) 21.00 (17.00 – 26.00) (N = 11)

Post-test 33.00 (30.00 – 36.00) (N = 13) 31.00 (27.00 – 35.00) (N = 11)

Follow-up 35.50 (30.50 – 38.75) (N = 8) 26.50 (21.25 – 37.00) (N = 4)

Diastolic blood pressure

Baseline 85.50 (78.00 – 89.00) (N = 12) 81.00 (79.00 – 86.00) (N = 11)

Post-test 83.50 (76.25 – 88.00) (N = 12) 77.00 (69.00 – 83.00) (N = 11)

Follow-up 78.00 (73.00 – 95.00) (N = 7) 73.50 (61.75 – 87.50) (N = 4)

Systolic blood pressure

Baseline 122.50 (115.75 – 147.50) (N = 12) 124.00 (115.00 – 137.00) (N = 11)

Post-test 121.00 (110.00 – 126.50) (N = 12) 121.00 (104.00 – 130.00) (N = 11)

Follow-up 108.00 (107.00 – 124.00) (N = 7) 108.50 (91.50 – 125.50) (N = 4)
